# Impact of asymptomatic detection of non-SARS-CoV-2 respiratory viruses on pediatric cardiac surgical outcomes

**DOI:** 10.1017/ash.2024.414

**Published:** 2024-10-01

**Authors:** Laura J. Fischer, Mark Castera, Hannah Chin, Junghyae Lee, Kari A. Simonsen

**Affiliations:** 1 Child Health Research Institute, University of Nebraska Medical Center, Omaha, NE, USA; 2 College of Public Health, University of Nebraska Medical Center, Omaha, NE, USA; 3 Children’s Hospital of Philadelphia, Philadelphia, PA, USA; 4 Creighton University, Omaha, NE, USA; 5 UPMC Children’s Hospital of Pittsburgh, Pittsburgh, PA, USA; 6 Department of Pediatrics, University of Nebraska Medical Center, Omaha, NE, USA

## Abstract

Preoperative nasopharyngeal viral screening may reduce clinical uncertainty of upper respiratory infections prior to pediatric cardiac surgery but with unclear benefit. From March 2018 to March 2020, patients aged <3 years were screened for respiratory viruses and had substantial rates of viral detection (40%) but no observed differences in outcomes.

## Background

Intraoperative and postoperative complications associated with symptomatic upper respiratory infections (URIs) in very young children are well documented.^1,2^ However, asymptomatic respiratory viral detection occurs frequently and may be prolonged.^3^ In the absence of clear clinical guidelines for pediatric preoperative evaluation of URIs, clinical examination typically determines the patient’s surgical fitness. Availability of multiplex polymerase chain reaction (PCR) testing for respiratory viral panels (RVPs) is an attractive option for preoperative screening; however, the value of an RVP’s diagnostic information should be balanced with increased costs and potential surgical delay.^1,4^ Diagnostic stewardship requires appropriate management of such diagnostic resources to maximize benefit and reduce waste.^5^

Community-based RVP screening shows 11–47% of asymptomatic children test positive via PCR for rhinovirus/enterovirus, the most common respiratory viruses identified via RVP.^3^ Rhinovirus takes up to 5–6 weeks to resolve from the nasal mucosa in children.^6^ When using RVP for screening, positive results may not clearly distinguish symptomatic infection, pre-infectious state, asymptomatic infection, or residual positive following infection resolution.

Pediatric patients requiring cardiac surgery may be at high risk for adverse clinical outcomes associated with respiratory viral infections, including risks of death, prolonged ventilation, and increased length of stay.^2^ The impact of RVP screening in addition to usual clinical examination for preoperative surgical clearance on patient interoperative and postoperative outcomes has limited supporting evidence.^1,2^

This study aimed to assess whether a positive preoperative RVP (+RVP) affected postoperative length of stay in the PICU or hospital and assessed for an association between +RVP and adverse clinical events in young, asymptomatic pediatric patients undergoing cardiothoracic surgery.

## Methods

Patients <3 years old presenting to a single children’s hospital for cardiothoracic surgery from March 2018 to March 2020 were enrolled. Patients were deemed asymptomatic for viral infections at time of enrollment by preoperative clinical examination. Informed consent was completed at preoperative clinic visit or within preoperative surgical area on surgical day. Within 24 hours prior to surgery, Biofire® RVP (not including SARS-CoV-2) via nasopharyngeal swab for PCR was collected from participants. Clinical laboratory performed the RVPs and faxed results to study investigator. The clinical team was blinded to results. Chart review was completed after hospital discharge for patient hospital course including demographics, length of stay, and postoperative adverse clinical events including ventilator-associated pneumonia (VAP), surgical site infection (SSI), postoperative fever (recorded temperature ≥100.4 °F), need for postoperative ventilation or re-intubation, antibiotics resumed >24 hours but <14 days postoperatively, and mortality. A sample size of 108 (36 in +RVP and 72 in control group) would achieve 80% power to detect a 20% difference between the two groups, assuming a significance level (alpha) of 0.05. The study was overseen by the University of Nebraska Medical Center Institutional Review Board. Statistical analyses were conducted with SAS® 9.4.

## Analysis

Frequencies were calculated for demographics. Mean difference between RVP status and length of stay for PICU and hospital was assessed with an independent, two-group, sample *t*-test. Logistic regression was used to test association between RVP status and adverse clinical events, adjusted by complexity of surgical procedure using the Risk Adjustment in Congenital Heart Surgery-1 (RACHS-1) score, scaled 1–6 with increasing risk. Fisher’s exact test was used to test association between RACHS-1 score and RVP result.

## Results

There were 111 participants enrolled with 18 excluded from analysis (14 screen fails, 2 invalid consents, and 2 extreme outliers with significant, ongoing medical complexity) for a total of 93 participants of which 51% were male, 75% Caucasian, with a mean age of 7.6 months (range: 0–33 months) (Table 1). Among enrolled participants, 40% had a +RVP (Table 1). Adenovirus, non-SARS-CoV-2 coronaviruses, rhinovirus/enterovirus, respiratory syncytial virus, and parainfluenza were the pathogens detected preoperatively with rhinovirus/enterovirus being most frequent. Among participants with a +RVP, 19% tested positive for more than one virus.

**Table 1.** Demographics, clinical characteristics, and outcomes in pediatric patients undergoing cardiac surgery

Variable	Frequency (N = 93)
Race	**n (%)**
African American/Black	2 (2.2)
American Indian	1 (1.1)
Asian	2 (2.2)
Non-Hispanic Caucasian	70 (75.3)
Hispanic	12 (12.9)
Indian	1 (1.1)
Two or more races	5 (5.4)
Sex	**n (%)**
Female	46 (49.5)
Male	47 (50.5)
Age	**Mean (Range)**
Months	7.6 (0–33)
RVP status	**n (%)**
Positive	37 (39.8)
Negative	56 (60.2)
Multiple versus single virus	**n (%)**
>1 positive	7 (18.9)
Single positive	30 (81.1)
Specific virus	**n (%)**
Adenovirus	8 (17.0)
Non-SARS-CoV-2 coronavirus	4 (8.5)
Rhinovirus/Enterovirus	30 (63.8)
Respiratory syncytial virus	4 (8.5)
Parainfluenza	1 (2.1)
RACHS-1 score	**n (%)**
1 (Lowest risk)	9 (9.7)
2	30 (32.3)
3	39 (41.9)
4	13 (13.9)
5	0 (0.0)
6 (Highest risk)	2 (2.2)
Clinical adverse event	**n (%)**
Postoperative fever	52 (55.9)
Antimicrobial therapy	16 (17.2)
Postoperative reintubation	6 (6.5)
Left OR extubated	75 (80.6)
NPPV	43 (46.2)
SSI	3 (3.2)
VAP	0 (0.0)
Mortality	0 (0.0)
Length of stay (days)	**Mean (SD)**
Hospital	9.3 (8.6)
PICU	4.6 (4.7)

Note: Non-SARS-CoV-2 Coronavirus, Coronaviruses 229E, NL63, or OC43; Antimicrobial Therapy, antimicrobial therapy restarted >24 hours but <14 days postoperatively; OR, operating room; NPPV, noninvasive positive pressure ventilation; SSI, surgical site infection; VAP, ventilator-associated pneumonia; PICU, pediatric intensive care unit

Table 1 also shows the RACHS-1 scores for patients, with a typical distribution of scores 2–3 being most common, and frequency of postoperative clinical adverse events including postoperative fevers (the most reported adverse event at 55.9%), SSIs, and respiratory status indicators including extubation before leaving operating room (OR), reintubation, and need for noninvasive positive pressure ventilation.

There was no statistical association between RVP status and length of PICU (*P* = 0.29) or hospital stay (*P* = 0.74) (Table 2), nor between RACHS-1 score and RVP status (*P* = 0.88). Left OR extubated with +RVP was statistically significant, but small sample size and broad confidence intervals limit the interpretability of this observation (Table 2). No other clinically or statistically significant associations RVP status and adverse events were observed.

**Table 2.** Differences in length of stay and clinical adverse events by RVP status*

Location	RVP status	N = 93 n (%)	Mean (days)	Standard error	*P*-value
Hospital	+RVP	37 (39.8)	8.92	1.42	0.74
	−RVP	56 (60.2)	9.54	1.15	
PICU	+RVP	37 (39.8)	4.00	0.77	0.29
	−RVP	56 (60.2)	5.05	0.63	

Clinical adverse event	RVP status	n (%)	Adjusted odds ratio	95% confidence intervals	
Left OR extubated	+RVP −RVP	36 (48.0) 39 (52.0)	19.58	2.25	170.66
Postoperative reintubation	+RVP −RVP	1 (16.7) 5 (83.3)	0.29	0.03	2.55
Postoperative NPPV	+RVP −RVP	16 (37.2) 27 (62.8)	0.84	0.34	2.09
Antimicrobial therapy	+RVP −RVP	8 (50.0) 8 (50.0)	1.72	0.57	5.23
Postoperative fever	+RVP −RVP	19 (36.5) 33 (63.5)	0.74	0.32	1.70
SSI	+RVP −RVP	3 (100.0) 0 (0.0)	12.17	0.61	242.84

Note: +RVP, positive RVP; -RVP, negative RVP; PICU, pediatric intensive care unit, OR, operating room; NPPV, noninvasive positive pressure ventilation; Antimicrobial Therapy, antimicrobial therapy restarted >24 hours but <14 days postoperatively; SSI, surgical site infection.

* No ventilator-associated pneumonia (VAP) or mortality events were reported in study sample.

## Discussion

This single-center study detected virus in a sizeable proportion of pediatric patients who were undergoing cardiac surgery within 24 hours of testing. The observed number of patients with viral detection was within the expected range for asymptomatic <3-year-olds.^7^ We were unable to identify any postoperative complications associated with viral detection that would have been missed by clinical symptom screening and physical examination alone. These findings support recommendations that preoperative RVP screening should be discouraged, even among high-risk patients with a high pretest probability for viral detection, such as this young pediatric cardiac surgical population. Rather, final surgical fitness should be deferred to the clinical team, based on patient’s medical history and physical examination.

Previous literature has advocated for preprocedural testing; however, many of these studies primarily focused on outcomes in symptomatic patients or test positivity, without delineating between symptomatic and asymptomatic patients.^1,2,8^ It is also important to note that test utilization for asymptomatic screening is against some manufacturers’ recommendations for their products.^8^

Several limitations should be noted for this study. The study was limited by the availability of patients at a single pediatric hospital who agreed to participate. Although severity of surgery was adjusted for with the RACHS-1 score, surgical team’s plan to extubate postoperatively was not directly known. Additionally, the study was slightly underpowered due to early termination and inability to replace ineligible participants at the onset of the COVID-19 pandemic, which led to a stoppage of all observational research studies in our facility and restrictions on elective surgeries. As such, results should be interpreted with caution and validated with larger studies. There was a significant shift at the start of the pandemic in the types and urgency of cardiac surgeries performed, along with an imperative to conduct preoperative COVID-19 testing as asymptomatic detection of SARS-CoV-2 could occur for 2 weeks, potentially affecting study hypothesis/outcomes.^9^ Going forward, SARS-CoV-2 may require different preoperative testing considerations and additional study compared with these respiratory viruses, especially in very young, SARS-CoV-2-naïve children with congenital heart defects.^10^

Future directions could include larger, multi-center investigation to explore whether specific pathogens, particularly COVID-19, play a role in postoperative adverse events, including prolonging LOS. Additionally, future studies could examine other impacts of postoperative patients’ viral detection, including respiratory transmission potential from asymptomatic patients in both OR and ICU settings.

This limited, single-center study suggests preoperative RVPs in young pediatric cardiac surgery candidates detected asymptomatic virus in 40%, though detection of virus provided no evident clinical benefit in a pre-COVID population. Larger studies are required to fully explore specific adverse clinical events, more effectively identify high-risk patients, and to detect differential impacts of specific respiratory viruses. Broad institutional policies requiring preoperative RVP screening for pediatric cardiac surgery in asymptomatic patients should be discouraged.

## Data Availability

None.
